# A multi-case study of the vertically integrated health-care at county-level in rural China: towards holistic and dynamic governance

**DOI:** 10.3389/fpubh.2023.1178179

**Published:** 2024-02-14

**Authors:** Wenhui Xu, Li Zhu, Zixuan Peng, Xu Chen

**Affiliations:** ^1^Institute of Civil Affairs and Social Work, Changsha Social Work College, Changsha, Hunan, China; ^2^School of Politics and Public Administration, Guangxi Minzu University, Nanning, China; ^3^School of Public Health, Southeast University, Nanjing, China; ^4^Zhejiang Provincial Philosophy and Social Sciences Pilot Laboratory, Hangzhou, China; ^5^Laboratory of Intelligent Society and Governance, Zhejiang Lab, Hangzhou, China

**Keywords:** vertically integrated health-care at county-level, health governance paradigm, collaborations of multiple actors, China, multi-case study

## Abstract

**Background:**

In contrast to the Grading Diagnosis and Treatment System (GDTS), Vertically Integrated Health-care at County-level (VIHC) is a strategic policy in rural China. This research intends to analyze the shift in governance paradigm with regard to the adjustment of the power structure and interest relationships among various participants, using the building of VIHC as a cut-in point.

**Methods:**

We carry out a multi-case study to investigate the paradigms of health governance when building VIHC in three different rural counties in China.

**Results:**

There were exchanges between government and other participants, vertical and horizontal collaborations among government divisions, and prompt responses to public requirements. County C’s local administration, in particular, placed a strong emphasis on bureaucratic power and collaboration between various departments both within and outside of administrative boundaries. In contrast, County B’s local administration emphasized the independence of healthcare practitioners and worked to win their support. In contrast to the previous two governments, County A encouraged social actors to participate and saw a little improvement in performance.

**Conclusion:**

In examining the health reform in rural China, this study paints a picture of the development of the health governance paradigm. In rural China, a comprehensive and dynamic governance paradigm was created through the integration of the health decision-making process, which was driven by the public’s health needs, the operation mechanism, which featured both competition and cooperation, and the action logic of sharing responsibility.

## Introduction

The concept of integrated healthcare was initially created in developed countries, such as the United States, Britain, Germany, Canada, etc. In the middle and late 20th century, these countries faced the problems of service decentralization and rising medical costs. To improve the utilization of resources, cut down on service costs, and increase patient satisfaction, the healthcare systems in Europe and the US underwent integrated reform. The era of integrative healthcare is only getting started (1).

In China, the development of county-level integrated healthcare can be divided into the following three stages (2):

Phase I: The establishment of rural integrated healthcare service delivery from 1949 to 1978. In this period, the national economy was facing recovery and reconstruction, and the lack of government financial resources contributed to the inadequate rural healthcare system. In order to change the aforementioned situation, governments at all levels reformed and built health institutions in the 1950s, gradually forming three-level (county hospitals-town health centers-village clinics) health and medical institutions in rural areas. The government places emphasized on the functional collaboration and labor division among the three levels of institutions (3). Coupled with the obvious political color in the period of planned economy, health departments at all levels formed a good cooperation mechanism. The three-level Grading Diagnosis and Treatment System (GDTS) has built in rural China. This system, with the primary health care facilities focusing on disease prevention and the hospitals focusing on disease treatment, is supposed to have tremendous strengths in distributing health resources and generating social benefits.

Phase II: Disruption and decentralization of comprehensive rural health services, 1979–2009. In the 1980s, the decrease of government financial support led to the collapse of GDTS (4, 5). The concept of primary health care services was watered down as a result of declining government financial support. The positioning of medical and health institutions were gradually blurred, and the competition of homogeneous services was fierce. Rural residents were faced with disordered medical treatment and fragmented service provision. As a result, the three-level rural health and medical service network faced disintegration. Meanwhile, the construction of the New Rural Cooperative Medical Care Insurance System released the suppressed rural health needs (6). As a result, people flocked to tertiary hospitals to seek for high-quality medical services, while primary care institutions were facing the crisis of shutting down.

Phase III: The gradual return of rural integrated healthcare service provision, 2009-present. As Chinese economic strength grows, the government is paying more attention to livelihood issues, including the health and well-being of rural residents. In order to solve the urgent issues, such as “difficult and expensive to get health and medical services”, the low level of primary health and medical services, the health department began to explore deepening the comprehensive reform of primary health and medical institutions. Integrating county health resources, improving the service capacity of primary health and medical institutions and delivering seamless health services was proposed on China’s health policy agenda. In 2017, The General Office of the State Council of China promulgated the Guiding Opinions on Promoting the Construction and Development of Integrated healthcare system, which clearly proposed the establishment of Vertically Integrated Health-care at County-level (VIHC). So far, the integrated service provision of rural three-level medical service network is being reshaped and improved.

In recent years, the pilot program of VIHC which aiming to ameliorate inadequacy of health services across rural regions and address the structural imbalance of resources between county-level hospitals and township-level health services providers has been experimented across Chinese rural regions (7). In practice, the county-level hospitals cooperate with township-level health services providers through promoting the standardization of medical services, achieving unified but limited management toward health resources or forming a health community linked by interests (8). Meanwhile, under the institutional configuration of bureaucratic marketization (9), local health authorities, which are also known as the local health commissions, have been creatively reforming their health systems and replicating successful practices of other districts. It follows then that policy practices of VIHC depended on the action strategies of multiple actors, interactions among these different actors could lead to the coexistence of structural similarities and inconsistencies, raising questions about power and interest structure between the government and other actors. Taking the construction of VIHC as a cut-in point, we could analyze the transformation of health governance paradigm concerning the adjustment of the power structure and interest relationship among multiple participators.

A number of attempts have been undertaken to identify the role of government and its interactions with other actors. Previous researches have suggested that the deregulation of the bureaucratic departments may serve as the possible explanations. Health services providers were struggling in integrating into a closely-connected interest community due to the lack of financial incentives and public visions (10). Therefore, in the long term, it is inevitable to abolish the government-led integrated health-care and allow the self-exploration of the health services providers (11). Other research, however, indicated that the increasing regulation of the authorities might be the answer. From this perspective, the lack of competition among health services providers has the potential to weaken the effects of VIHC, impair the quality of health services, and reduce the efficiency of health care institutions. Hence, it is necessary to strengthen government control and reduce health services providers’ desire toward interests (12). Though with obvious differences, both the above two arguments focus on the role of government and its interactions with other actors. Recently, different health services providers have emerged in the Chinese health market, and it has been agreed that the health system reform cannot be promoted solely by the government departments (13). A study also pointed out that the governance paradigm in the construction of VIHC has to be transformed from the mutual opposition between government and other players to the interactions among government, market participators and social players in this new era (14). However, the actual internal mechanism among these multiple actors has not yet been explored by prior studies (10, 15).

Our study aims to explore the interactions among different parties when practicing VIHC in China’s rural area. In this study, we will depict the diachronic evolution of health governance paradigm under China’s authoritarian environment through looking at how the Chinese government implement the policy practice of VIHC. Subsequently, a new theoretical framework will be presented and possible suggestions will be derived based on the research findings.

## Materials and methods

### Theoretical basis

In the context of China’s health system reform, the VIHC aims to create an effective hierarchical diagnosis and treatment pattern by building a collaborative relationship between the three-level health and medical institutions (county hospitals, town health centers, and village clinics) (16). The construction of the VIHC involves a large number of participants with different development goals, interest demands and behavioral motivations. These participants have formed a complex interacting relationship with various dimensions, numerous levels, and mutual embedding under the influence of external factors such as system and social context. Therefore, it is necessary to discuss the construction of the VIHC from governance perspective. Governance theory addresses to explain the interaction mechanisms among multiple actors across regional, domain and organizational boundaries in their cooperation to solve intractable problems (17).

New Institutionalism theory regards health system reform as a continuous interaction process between health services providers and the institutional environment. More specifically, the institutional environment provides paths for organizational behaviors and influences the evolution of governance paradigm (18–20). Meanwhile, hospitals are complex organizations and highly dependent on the institutional environment (21). The Institutional Analysis and Development framework was then proposed that health system reform was largely depended on the potential influence of a series of exogenous variables on health governance practices (22, 23). Based on the above theories and the corresponding views, we claim that multiple actors interact in the certain institutional environment, and their actions are affected by both exogenous and endogenous variables. Therefore, a new theoretical framework remains to be proposed to explain the transformation of health governance paradigm in rural China.

### Case study design

Though the institutional arrangement provides fundamental paths, health governance paradigms vary by region and time. In our study, the transformation of the health governance paradigm will be reflected through the change of power structure relationship during interactions among multiple actors. To depict the diachronic evolution of health governance paradigm characterized by diverse and complex factors, the case study is appropriate for use (24).

Three cases were selected to satisfy the replication requirements of performing a multi-case study (25). Sample counties have to satisfy the criteria of reconstructing the grading health system and practicing VIHC for at least 3 years. In general, Chinese eastern provinces are usually chosen as the pilot places to implement health policies and their experiences will then be referred by central and western provinces. In this sense, the development degree of the three counties was quite different. The specific characteristics of sample cases are shown in Table 1. The three regions had similar governance structures prior to reconstructing the grading health system and implementing VIHC: in terms of finances, there is a separation between revenue and expenditure; in terms of human resources, the health administrative departments have the power of appointment and removal of personnel of primary health centers. Similar governance structures lead to common problems in the three regions: rigid governance structures and low grassroots enthusiasm.

**Table 1:** Characteristics of sample cases.

VIHC	City	Province	Location	Pilot area	GDP (Million)	Start of reform	Data availability
A	Zhanjiang	Guangdong	Eastern	Yes	24,714.86	2015	Yes
B	Changsha	Hunan	Central	No	143,273.54	2016	Yes
C	Nanning	Guangxi	Western	Yes	5674.93	2014	Yes

Source: the authors collected the data through government information disclosure process.

To better explain the actual internal mechanism among these multiple actors, the effectiveness of VIHC was embedded in our case study. Theoretical, the effectiveness of the community-based network could be assessed at three levels: the community-level, the network-level, and the organization-level (26), and the community-level assessment could reflect the results at the organizational and network levels and is often the best choice (27). In this research, we compared the effectiveness of the community-level VIHC with the required diagnosis rate at 90%. Specifically, the effectiveness of VIHC were measured by the triple aim indicators, including the growth rate of outpatient visits of primary health institutions, the growth rate of referrals from county-level hospitals to primary healthcare institutions, and annual growth rate of patient visits.

### Data collection and analysis

Table 2 provides details of the data collection methods. Our research adopted 3 ways to collect data: First, first-hand data were obtained via conducting 16 semi-structured interviews from September 2018 to June 2019. Among these interviews, 5 were officials of the health department, 10 were the staff of healthcare institutions, and 1 was the representative of social player (Table 3). Interview outlines were prepared before the formal interview: as for officials, questions mainly concerned their views on the construction of the integrated healthcare networks at county-level, the distribution of health resources, the construction of institutional environment and the innovation of the health system; as for health services providers, questions primarily involved supports obtained from various channels, their authorities over the allocating resources, and their interactions with the local health department. Second, supplementary policy documents, working reports and administrative data were also collected. Specifically, we firstly examined the publicly-issued policy documents regarding VIHC construction and then we reviewed work reports published by other actors. Third, more information was collected through observation and focus group discussion. After all the related research materials were collected, we performed a thematic analysis to code and analyze interview texts. All of the research results were verified through comparing research materials collected from different sources.

**Table 2:** Data collection methods.

Methods	Content	Reasons
In-depth interviews	Interviews with officials of the health departments, health services providers and representative of social players.	To reflect the policy process of VIHC and gain a deeper understanding of policy practice from different perspectives.
Documents	Public policy documents, work reports and administrative data.	To understand the formal policy process of VIHC in the three sample counties.
Observations	Informal observations in VIHC institutions.	To investigate how VIHC was implemented in institutions.
Focus group discussion	Group discussions about the policy practice of VIHC.	To identify and verify conclusions and seek for solutions to promote the development of VIHC.

**Table 3:** Information of interviews.

Respondent	Working place and role	Interviewing time
R 1	A county, Officials of the health department	February 27, 2019
R 2	B county, Officials of the health department	July 21, 2019
R 3	C county, Officials of the health department	October 30, 2018; March 1, 2019
R 4	B county, Dean of hospital	July 1, July 21, 2019
R 5	A county, Dean of hospital	February 27, 2019
R 6	C county, Dean of hospital	October 30, 2018; March 1, 2019
R 7	B county, Vice-dean of hospital	July 1, July 21, 2019
R 8	B county, Manager of VIHC	July 1, July 21, 2019
R9,R10,R11,R12,R13	B county, Primary health services providers in VIHC	July 21, 2019
R14	A county, Primary health services providers in VIHC	February 27, 2019
R15	C county, Primary health services providers in VIHC	October 30, 2018; March 1, 2019
R16	B county, Representative of social force	July 1, July 21, 2019

All the interviewees are anonymized.

## Results

### Results of the triple aim

From 2015 to 2016, the growth rate of primary annual patient visits and outpatient visits in county B were the highest (8.80 and 2.82% respectively) among all counties. From 2016 to 2017, county B had the lowest annual growth rate of patient visits (−3.14%), whereas it also had the highest increasing rate (3.64%) in primary outpatient visits. During the same period, the growth rate of referrals from county-level hospital to primary healthcare institutions in county C was 19.78%, while the other two counties showed a substantial decreasing trend (Table 4). These results were embedded in qualitative materials to contribute to the comprehensive analysis.

**Table 4:** Implementation results.

VIHC	Growth rate of outpatient visits in primary health institutions		Growth rate of referrals from county-level hospitals to primary health institutions		Annual growth rate of patient visits	
	2015–2016	2016–2017	2015–2016	2016–2017	2015–2016	2016–2017
A	−11.43%	0.73%	70.59%	−86.21%	−8.35%	4.07%
B	2.82%	3.64%	4.84%	−63.08%	8.80%	−3.14%
C	0.75%	−4.89%	47.03%	19.78%	5.28%	1.51%

### Behaviors of government

As shown in Table 5, compared with the other two counties, both the organizational and financial support received by county B was relatively lower. County C initiated VIHC earlier than county B when the subsidy policies for county-level hospitals and township-level health services providers had not been proposed and all the health services providers were unwilling to embrace VIHC. To implement policy practice of VIHC, the head of the whole government and related departments in county C, including the health department, the human resources and social security department, the financial department and the like, organized investigations and made an implementation plan to allocate human resources and health funds. As a result, county C had better performance in realizing the triple aims. These results indicate that (1) Compared with county B where the single health department took the responsibility to implement VIHC, the whole government in county C had stronger strength in attracting organizational and final support (as shown in Table 5); and (2) horizontal interactions among different government departments were found during the VIHC practices in county C.

**Table 5:** Governmental support.

County	Organizational support	Financial support
A	A team led by the head of county-level government was established to promote coordination among the health department and other departments.	In 2014, medical and health expenditure valuing at ¥ 23.2 million was used to promote public health equality and public hospital reform; The special financial fund to construct VIHC was about ¥ 2 million.
B	A team led by the head of county-level heath department was established.	In 2017, the finance department provided ¥ 13.1 million for health reform; Financial support for the construction of VIHC was about ¥ 0.75 million.
C	A team led by the head of county-level government was established; The Health Integration Mobilization Conference organized by the Chinese Peopl”s Political Consultative Conference was held.	Over ¥ 10 million per year was used to update infrastructure and medical equipment of health services providers at the county- and township-level; Fiscal support for the construction of VIHC was also provided.

Sources: authors organized the table according to policy documents and work reports.

*The primary challenge In this region is that other relevant administrative departments do not understand the significance of implementing VIHC and did not provide enough support*. *(R4 who was the chief of county-level hospitals said on the round table meeting organized by the head of the county health department in County B).*

*The county-level hospital is difficult to recruit new staff, let along to have new medical personnel, and administrative measures are necessary during the early years of health reform*. *(R3 who was an official from the health department in county C said during the interview).*

In order to practice VIHC, supported by the whole government in county A, a panel group consisting of officials from the health department and related departments investigated the implementation environment in 2016. They found that the shortage of health services resources and inadequate capacity of township-level health services providers were the major issues faced. Meanwhile, the previous fiscal system stipulated that the income and expenditure of the healthcare institutions were managed by the government solely. This curbed health services providers’ enthusiasm to increase the value for money and improve the quality of services. To address these problems, both the personnel system and the financial system were reconstructed in County A. Subsequently, these proposals were further accepted by the provincial government and contributed to the health system reform of Guangdong province. In 2017, the People’s Government of Guangdong Province (PGGP) published a provincial implementation plan to allow for abolishing the previous fiscal system and to encourage the municipal government to set special funds to facilitate VIHC (28). These results indicate the following implications: (1) Same as county C, county A received strong organizational and financial support (Table 5). The reason may lie in that instead of only being led by the single health department in B, VIHC practice was supported by the head of the whole government in county A and county C; and (2) Compared with county C, not only the horizontal cooperation but the vertical interactions among governmental departments were shown in county A during the policy practice of VIHC.

*Mandatory administrative policies published by the health department are not enough to support the development of VIHC, and therefore corresponding efforts from the whole government are needed*. *(R1 from the health department and R5 in the health services providers in County A said during the interview).*

### Behaviors of health services providers

Non-profit county-level hospitals and township-level health services providers within one administrative county were firstly involved in practicing VIHC. Specifically, in the three sample counties, the county-level hospitals provided guidance and support to township-level health services providers to jointly construct the integrated healthcare networks. Generally, a committee consisted of different health services providers was established to guide the standardization of diagnosis/treatment services and manage the human, financial as well as equipment resources (Table 6). In detail, the county-level hospitals in county A and county C were delegated and empowered by the health authorities to allocate human resources in VIHC and this strategy reformed the previous personnel management system. Moreover, in county A, a finance and drug management center was also established to distribute medical resources among the county-level hospitals and township-level health services providers. In the meantime, the county-level hospital who was often leading actor and the member institutions contracted an agreement on the responsibility of medical accidents. Specifically, the county-level hospital was responsible for medical accidents occurred in member organizations and had to undertake 10% of the expenditures of medical accidents. In contrast, expert councils in county B were unable to manage health services providers’ human and medical resources, and county-level hospitals in county B was not involved in sharing responsibility with other health services institutions. Instead, they only focused on sharing examination equipment and diagnosis information.

**Table 6:** Self-exploration of each VIHC.

VIHC	Organizational settings	Composition of members	Explanation
A	Management Office	Mainly included staff members from the county-level hospital.	An office was established to standardize medical business and achieve unified but limited management of human, medical and equipment resources.
B	Expert Council	Members from all the health services providers.	Councils were built to guide the construction of distinctive clinical specialists in health services providers and realize the win-win cooperation.
C	Management Office	Mainly composed by the staff members from the county-level hospitals.	An office was established to standardize medical business and implement unified but limited management of human, medical and equipment resources.

Sources: authors organized the table according to policy documents and work reports.

*In terms of improving the capacity of primary-level medical and health services during the policy practice of VIHC, medical assistance from county-level hospitals are very useful and the goal of implementing win-win strategy through discussion and collaboration is beneficial*. *(R9/R10/R11/R12/R13 from primary health care providers in county B, R14 from primary care providers in county A and R15 from primary health care providers in county C said during the interview).*

Certainly, the county-level hospital may also compete with township health services providers as the functions of the county-level hospitals partially overlapped with functions of township health centers. Practically, the county-level hospitals owned more diagnosis and treatment equipment and had advantages over township health centers, therefore, we could also find competition between county-level hospitals and township health services providers across the three counties.

### Behavior of other players

In this research, other players refer to hospitals not located in certain county area, private health providers and non-government health promotion organizations. In our research, the participation of these actors also exerted influence on promoting VIHC. Specifically, as for county B, the hospitals not within county B and private health providers were permitted to connect township-level health services providers to form diverse kinds of health care alliances, thus improving service capabilities of primary health services providers to some extent. As for county C, health services capacity was insufficient and other forms of health care alliances were also shown. Municipal-level hospitals provided medical assistance to health services providers and formed an administration-oriented coalition which is not sustainable in the long run due to the lack of inner incentives; Meanwhile, the leading hospital in county C gained support from a tertiary hospital in Gaozhou city, making full use of the policy of medical aid. As for county A, a private hospital was supported by the local government to satisfy the increasing health needs of residents; an information technology company was also involved in constructing an artificial intelligent audit system to normalize the use of medical insurance funds across different health care institutions. This implies that private heath actors were emerging and their influences could not be ignored:

*We were involving in the construction of the information system to connect the leading hospital and the primary health services providers. (R16 who is the staff of a private company in county B said during the interview).*

## Discussion

### Interpretation of findings

As the multi-case analyses illustrated, the specific strategies to implement VIHC vary by region and time. County C’s administration placed a strong emphasis on maximizing bureaucratic influence and encouraging cross-departmental collaboration. Government in county B, in contrast, gave health service providers more room to operate, attempting to strike a balance between governmental supervision and self-discovery. Contrary to earlier norms, county A’s entire county government adopted VIHC by encouraging societal forces to participate.

Different implementation results were found in the three sample counties, and the continuous increase in both the annual growth rate of primary outpatient visits and the annual growth rate of patient visits was to be expected considering the efforts of the local government. However, the expected positive results were only found in county A, suggesting that the policy practice of VIHC with cross-department, cross-level and cross-section cooperation laid a solid foundation for the successful adjustment of the power structure relationship. Specifically, the government played a critical role in constructing VIHC, as the health department and related departments affected the behavior of public and private health services providers. Moreover, a balanced relationship between the authority of the government and the independence of the health services providers helped to enhance the implementation results.

The policy practice of VIHC in county B and county C as well as their implementation results could explain that either the lack of administrative support or the absence of social participation would contribute to the undesired implementation results. As for county B, we found that it was difficult to enhance the annual growth rate of patient visits than the other two counties, and insufficient governmental support provided a possible explanation. Specifically, the head of county-level heath department rather than the head of the holistic government in county B brought limited administrative and financial support. We also found that the growth rate of primary outpatient visits was increasing, whereas the annual growth rate of patient visits showed the opposite trend from 2015 to 2017. This inconsistency could be partially explained by the strategies of municipal-level hospitals and the influence of residents’ behavior pattern of seeking health services. Specifically, county B is adjacent to Changsha city (the capital city of Hunan province) and its residents were more inclined to visit the municipal-level hospitals in Changsha city.

As for county C, it is found that the former indicator was declining and the latter indicator only slightly increased during the research period. As County C is located in an underdeveloped area and a great number of practitioners and assistant practitioners flew into developed western cities. Therefore, primary health care institutions in county C could hardly satisfy the needs of the residents, and the fragmented health network was unable to provide proper health services. To solve these problems, head of county-level government aimed to firstly improve the health services capability of the primary health institutions, and this strategy did bring positive effects at the early stage of the policy practice. However, the implementation results from 2016 to 2017 is desired, which may reveal that the health system reform under strict administrative control was already difficult to sustain.

To summarize, interactions of multiple actors were found when constructing VIHC. The actions of the government, the activities of the suppliers of health services, and the involvement of other stakeholders all had an impact on how successfully VIHC was implemented. In addition, the always shifting environment necessitates new health governance structures, which is in line with institutionalists’ perspectives that highlight the variety of organizational behaviors in various institutional environments (18, 19, 29).

### Developments in the construction of healthy county

Developments were demonstrated in constructing VIHC: First, the health system reform reflected by the construction of the VIHC was being conducted by the whole government, rather than the single health administrative department. Nowadays, providing health services in an ever-changing environment is not only the duty of the single health department but also the responsibility of the whole government (30). This research also found that all of the above three counties tried to obtain administrative support and financial support from the whole government. Second, the action logic of these different actors was heading toward multi-agent cooperation governance. As stated earlier, as a public issue, the health problem requires increasingly broader cooperation (13), and the administrative driven governance model is no longer relevant for VIHC’s ongoing development. In other words, the government’s policy goal of building VIHCs can only be accomplished with the help of the entire community. Third, the multi-agent cooperation governance centered by the government was steadily formed. Facing the challenges of the defragmentation of medical services and decentralization of responsibility, the current governance paradigm is refocusing on the active role of government (30). In this research, governance paradigm in the health sector is heading toward “the whole governance” or “the holistic governance.” This new health governance paradigm concerns more about the cooperation among multiple players and the timely response to the whole society. In conclusion, the vertical and horizontal collaboration between governmental departments, interactions of the whole government, health services providers and other participators, and their timely response to the needs of the public were found by this research.

### Steps ahead toward holistic and dynamic governance

**Figure 1:**
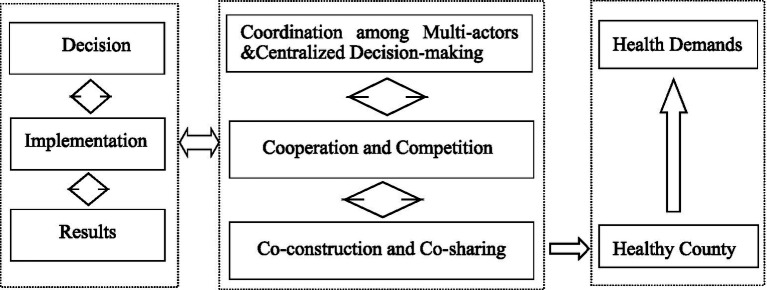
Holistic and dynamic framework.

Three key suggestions are provided for reference: (1) It is suggested to consider the opinions of multi-players to embed health in all policies at the early decision-making period. Better implementation results could be achieved by fully utilizing the potential of these multiple actors, (2) Both competition and cooperation could be promoted in the construction of VIHC. Competition and cooperation were established and maintained in China’s institutional framework for bureaucratic marketization, as previous study had shown (31, 32). The reason for stressing competition and cooperation lies in the goal of promoting high-quality health services and increasing value for money, (3) It is also suggested to promote continuous interactions among government authorities, health services providers and social participants. With the steps shown above, the holistic and dynamic framework featured with the integrated health decision-making process guided by the health needs of the public, the operation mechanism centered with competition and cooperation, and the action logic of co-sharing responsibility, is proposed (Figure 1).

### Study strengths and limitations

This study has several key strengths: To the best of our knowledge, this is the first paper to examine the actual internal mechanism among these multiple actors through conducting empirical research on VIHC in rural China. Besides, the multi-case study is more appropriate over other methods in addressing the complicated and exploratory questions proposed above. However, we should also recognize the limitations of this study: the first one is that the samples of this research are limited, which may impair the representativeness of the research findings; and the second is that we could only investigate the short-term implementation results, while it takes time for VIHC to fulfill its potential.

## Conclusion

Health governance paradigm has changed across almost all the countries and regions, including rural China. However, it is less clear about the diachronic adjustment of the power structure and interest relationship. We demonstrated the evolution of the interaction patterns among multiple actors and the existence of a holistic and dynamic governance structure in rural China. Specifically, the whole government at the county-level had played an important role in constructing VIHC, as it is powerful in attracting sufficient organizational and financial support. According to Japanese researcher Fukuyama, post-development nations should adhere to the state-led growth paradigm. This research can provide experience reference for the development of health service in developing countries. Besides, health services providers are also important in promoting the concept of healthy county. Their self-exploration of internal interest relationship with the government could contribute to the long-term effective governance of VHIC. Furthermore, the influence of other actors deserves more attention. Based on these facts, the holistic and dynamic governance structure concerning integration of decision, competition and cooperation in operation, and combination among different actors, may provide a possible theoretical basis for further research.

## Data availability statement

The raw data supporting the conclusions of this article will be made available by the authors, without undue reservation.

## Ethics statement

Ethical approval was not required for the study involving humans in accordance with the local legislation and institutional requirements. The studies were conducted in accordance with the local legislation and institutional requirements. Written informed consent to participate in this study was not required from the participants in accordance with the national legislation and the institutional requirements. The participants provided their verbal informed consent to participate in this study.

## Author contributions

WX, LZ, and ZP drafted the manuscript and contributed to the materials and analyses. LZ, ZP, and XC conceptualized and designed the study. All the author revised the manuscript. All authors contributed to the article and approved the submitted version.

## Conflict of interest

The authors declare that the research was conducted in the absence of any commercial or financial relationships that could be construed as a potential conflict of interest.

## Publisher’s note

All claims expressed in this article are solely those of the authors and do not necessarily represent those of their affiliated organizations, or those of the publisher, the editors and the reviewers. Any product that may be evaluated in this article, or claim that may be made by its manufacturer, is not guaranteed or endorsed by the publisher.
